# Microstructural evolution during sintering of copper particles studied by laboratory diffraction contrast tomography (LabDCT)

**DOI:** 10.1038/s41598-017-04742-1

**Published:** 2017-07-12

**Authors:** S. A. McDonald, C. Holzner, E. M. Lauridsen, P. Reischig, A. P. Merkle, P. J. Withers

**Affiliations:** 1 Henry Moseley X-ray Imaging Facility, School of Materials, University of Manchester, Manchester, M13 9PL UK; 2 Carl Zeiss X-ray Microscopy, Inc., 4385 Hopyard Road, Suite 100, Pleasanton, CA 94588 USA; 3 Xnovo Technology ApS, Galoche Alle 15, 4600 Køge, Denmark

## Abstract

Pressureless sintering of loose or compacted granular bodies at elevated temperature occurs by a combination of particle rearrangement, rotation, local deformation and diffusion, and grain growth. Understanding of how each of these processes contributes to the densification of a powder body is still immature. Here we report a fundamental study coupling the crystallographic imaging capability of laboratory diffraction contrast tomography (LabDCT) with conventional computed tomography (CT) in a time-lapse study. We are able to follow and differentiate these processes non-destructively and in three-dimensions during the sintering of a simple copper powder sample at 1050 °C. LabDCT quantifies particle rotation (to <0.05° accuracy) and grain growth while absorption CT simultaneously records the diffusion and deformation-related morphological changes of the sintering particles. We find that the rate of particle rotation is lowest for the more highly coordinated particles and decreases during sintering. Consequently, rotations are greater for surface breaking particles than for more highly coordinated interior ones. Both rolling (cooperative) and sliding particle rotations are observed. By tracking individual grains the grain growth/shrinkage kinetics during sintering are quantified grain by grain for the first time. Rapid, abnormal grain growth is observed for one grain while others either grow or are consumed more gradually.

## Introduction

The elevated temperature sintering of loose or compacted granular bodies towards full density is an important step in the production of engineering components having complex shapes via the powder metallurgical route. Unsurprisingly, sintering has a strong influence on the final microstructure and properties of a component^1^. The process is accompanied by densification of the body associated with changes of internal pore structure as the number of inter-particle bonds increases and their growth proceeds. Motions such as translation and rotation of particles have been shown to be important in contributing to the early stages of sintering^2, 3^ together with the development and growth of necks between neighbouring particles. Another important process is consolidation by interfacial and bulk diffusion leading to changes in pore morphology. Unfortunately this step can require long sintering times to eliminate large pores. This can lead to excessive grain growth^4^, which can have a detrimental effect on the performance of the final component, especially for ceramic compacts. Consequently, it is important to quantify and understand the extent to which these different mechanisms occur during consolidation so as to achieve nominally full densification with limited grain growth, for optimal performance.

For non-ideal arrangements of particles, in real systems of randomly packed powder bodies, irregular local and bulk movements are likely to take place during the very early stages of sintering. Local forces arising from local particle configurations can cause rotation and translational movements at an angle to the line connecting the particle centres^2, 3, 5^. Such particle rearrangement has been observed for cases of irregular packing in one-dimensional rows and two-dimensional planar layers of spherical particles^3, 6–9^. Microscopically, the main driving force for material transport during sintering is the reduction, and ultimately the minimization, of excess surface free energy^10^. To achieve this, geometric changes of the solid/porosity interface take place and transport of material into the pore occurs due to diffusion, particle rearrangements and plastic deformation. The possible diffusion pathways to/from the surface are bulk/volume diffusion, grain boundary diffusion or surface diffusion^4, 5^. Macroscopically, the result is a densification of the body towards full density as a result of reducing total pore volume^11^.

Concerning the contact region between neighbouring particles, the local chemical potential of atoms on, or close to, an external surface is a function of the surface curvature at the particle contact. Gradients in the lattice vacancy concentration are then responsible for transport of atoms via any of above diffusion paths, resulting in the growth of the contact area (neck) between the two particles. Excess vacancies are created at or immediately adjacent to the neck surface, migrate to the contact grain boundary and are annihilated. This sintering mechanism describes a continuous and homogeneous shrinkage (densification) by a centre-to-centre approach of two adjoining particles along their connecting lines^5^.

Classical absorption X-ray computed tomography (CT) has made a significant contribution to the quantitative study of sintering processes^12^. More recently, X-ray microtomography and digital image correlation (DIC) have been combined to evaluate the macroscopic strain fields associated with heterogeneous movement of regions of particles within a powder body and to link to the resulting non-uniform densification that occurs during powder transfer and compaction within a die prior to sintering^13–16^. Since conventional absorption tomography cannot reveal the intrinsic rotations of spherical particles that occur during the early stages of sintering, Grupp *et al*.^2^ and Kieback *et al*.^17^ used a method of drilling 8 micron sized marker holes into the surface of each of the spherical copper particles by focused ion beam milling. They then used *in situ* synchrotron X-ray microtomography to collect a continuous set of tomography scans. In following the evolution of the positions of the marker holes in three-dimensions the changes in rotation angle were measured with respect to a particular particle centre between sintering steps. Particles in the very early stages of sintering were observed to rotate around their own centres, becoming the dominant mechanism of particle movement and concluding that contact grain boundary sliding occurs. Several studies have quantified changes in particle and pore morphology during densification, using conventional absorption microtomography to study densification, neck size, particle coordination and pore distribution^12, 18–20^. In combining *in situ* synchrotron X-ray microtomography and 3D digital volume correlation during continuous acquisition McDonald *et al*.^21^ performed a volumetric strain analysis of a sintering powder body, indicating heterogeneities in straining associated with local non-uniform movement and densification of small aggregates of particles between which significant changes in shrinkage occur.

It is clear that both to better understand the contributions of particle rotations and diffusion/deformation to densification of a powder body and also to provide important insights into grain growth, it would be desirable to be able to map the constituent crystalline grains. The technique of X-ray diffraction contrast tomography (DCT) using monochromatic synchrotron^22–24^ has been shown to be capable of mapping crystal grains and their orientations non-destructively in three-dimensions. The DCT technique has the advantage that access to both the three-dimensional crystallographic information *and* the sample’s microstructure is provided. It has been used previously to provide temporal information about grain reorientation taking place during the early stages of sintering^25^ delineating the grain shapes and following changes in crystallographic orientation of individual crystal grains. Further, the recent development from monochromatic synchrotron to polychromatic laboratory-based X-ray DCT (LabDCT^26, 27^) promises wider accessibility of the DCT technique^28^ for in-depth studies of temporal changes in crystallographic grain structure non-destructively during ‘4D’ studies of microstructure evolution.

Here we use the newly established LabDCT technique to follow, non-destructively and in three-dimensions, the processes occurring during sintering of idealized copper powder for the first time. An initially loose collection of copper particles contained within an evacuated glass phial is sintered at 1050 °C for a total time of 420 min and the progress of sintering monitored by interrupting the sintering process at different stages to undertake absorption CT and DCT in a time-lapse manner. Conventional absorption CT is used to delineate the diffusion and deformation-related morphological changes of the sintering particles, while the particle rearrangements and rotations as well as the grain growth are simultaneously quantified by LabDCT. Such information provides unique insights into the sintering micromechanisms and grain growth and promises to contribute to better sintering process control leading to dense products with controlled grain growth in the future.

## Results

### Morphological changes

**Figure 1:**
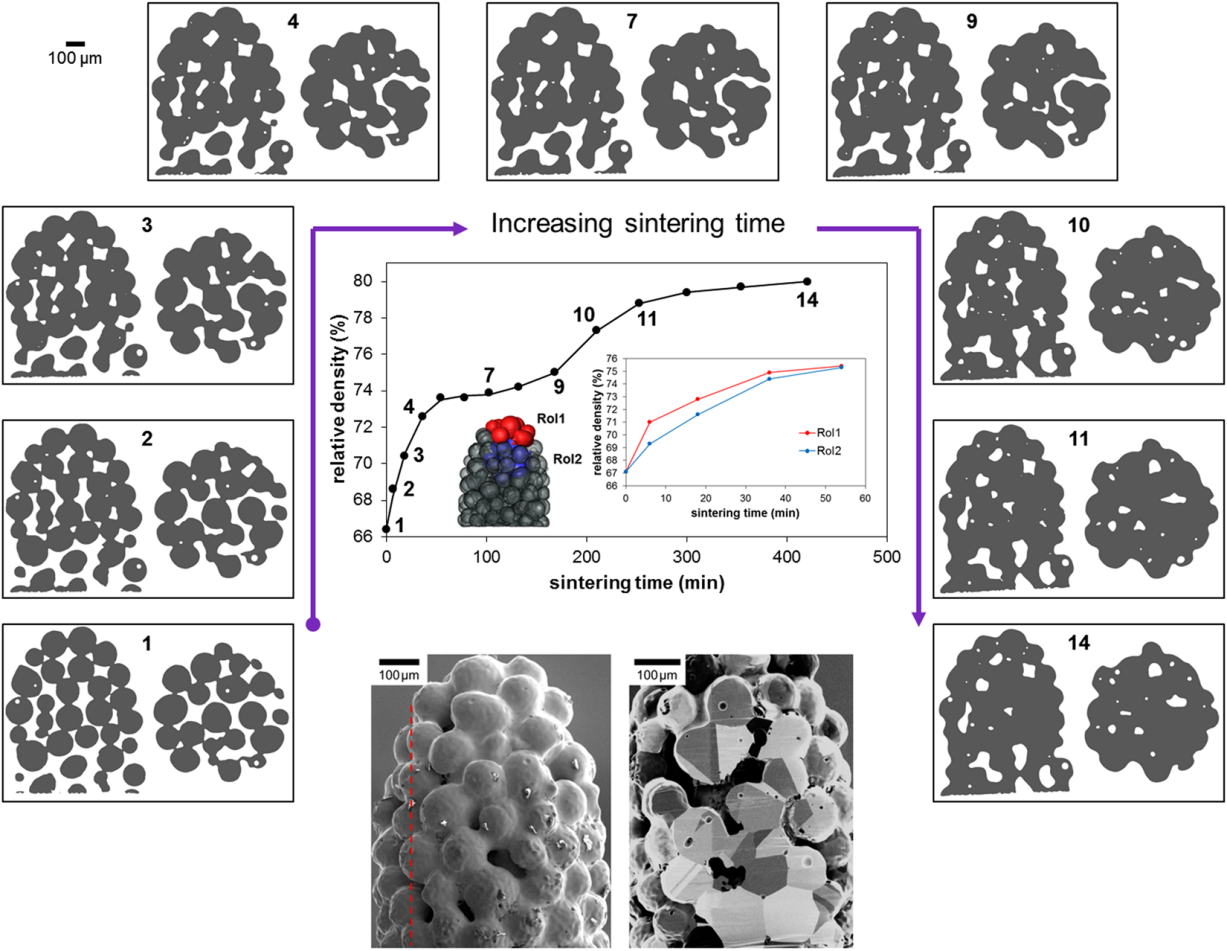
Densification of the powder body across the sintering steps at 1050 °C. Centre: Plot showing the change in density for the full sample and, inset, comparing two regions of interest (RoI) for the first 5 steps. The locations of these local RoI’s with respect to the sample are indicated in the 3D surface rendering (semi transparent) of the full sample; one from the top of the sample (RoI1, red) and one from within the centre of the sample (RoI2, blue). Shown alongside are pairs of virtual slices (left: vertical and right: horizontal slices) from the 3D renderings for selected steps reconstructed from the absorption tomography. A FIB-milled section of the final sintered sample is shown below-right, and its location in the sample is indicated by the dashed red line in the SEM image of the full sample (below-left).

Equivalent virtual absorption CT sections, both vertical and horizontal, for a region of the sintering Cu particles in the sample are shown in Fig. 1 for selected steps through the sintering process. As expected the early steps show the establishment and growth of inter-particle contacts, or ‘necks’, between neighbouring particles. As sintering progresses the sample densifies and the particle morphology observed in the early stages densifies progressively through decreasing pore volume. These changes result in an increase in the relative density of the powder body over time from 65% as a loose compact to 80% dense, as shown in Fig. 1. Unsurprisingly, the rate of densification decreases as sintering time progresses. The FIB section in Fig. 1 reveals the grain structure in the sample at the end of the sintering study. This cross-section indicates a grain size of ~70 µm after the final sintering step.

### Particle rotations and translations

**Figure 2:**
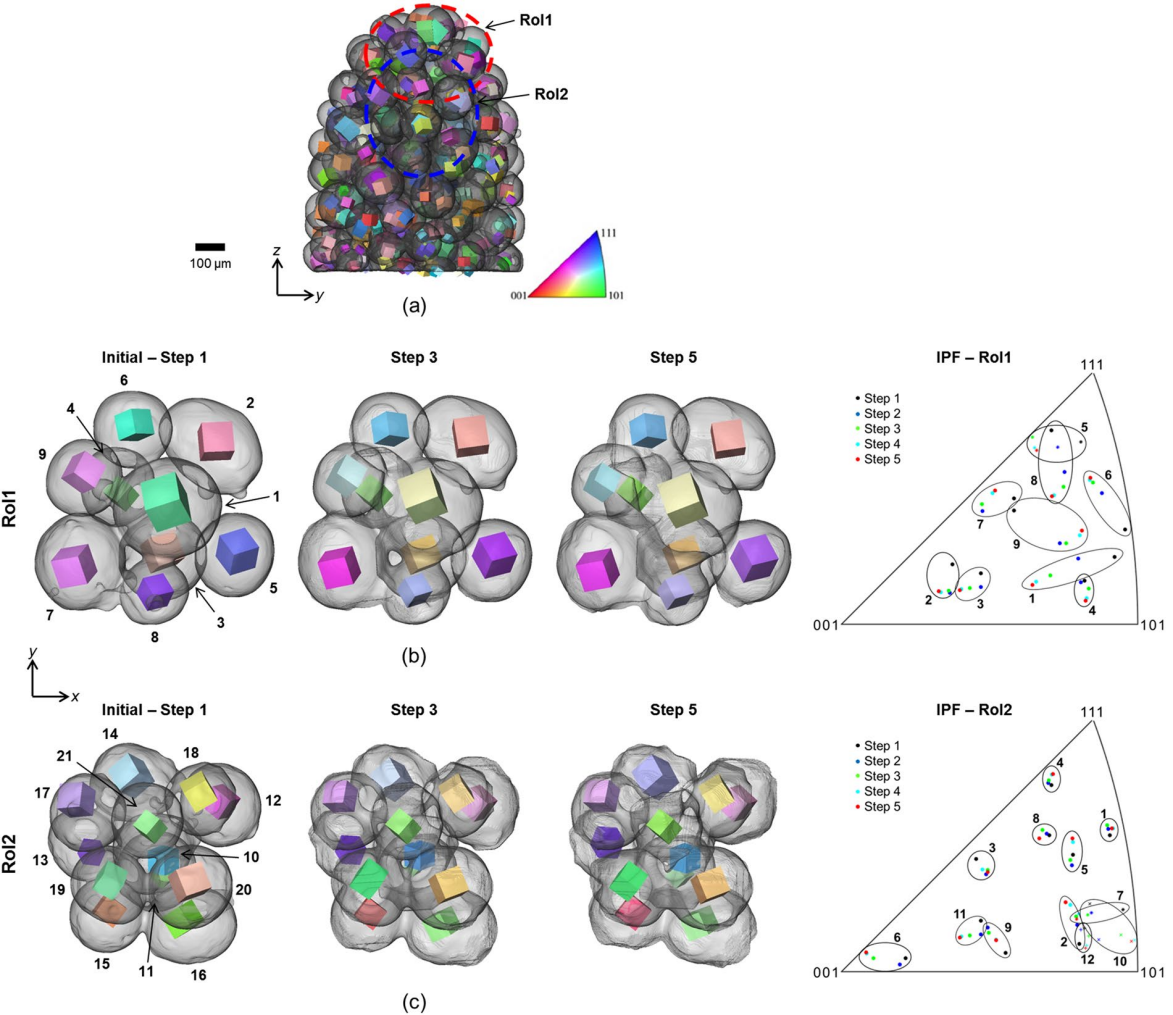
Particle rotations extracted from changes in crystallographic grain orientation during sintering at 1050 °C. (**a**) The absorption CT volume (transparent black and white 3D surface) of the copper particle sample and the associated LabDCT grain map reconstruction, for the initial sintering step. Crystal grains from the LabDCT analysis are represented as cubes centred on their measured (x,y,z) positions, revealing their crystallographic orientation (by colour according to inverse pole figure). The cubes are scaled by 0.5 according to the respective grain size. (**b**), (**c**) Two plan view image sequences showing the two local regions of interest (clusters, RoI1 and RoI2) have been extracted for further analysis; their locations with respect to the sample are indicated in (**a**) and in Fig. 1. The grains in each RoI are also shown plotted on an inverse pole figure (colour according to the IPF colourmap in (**a**)) to show their angular movements.

Application of the LabDCT technique enables particle rotations to be measured and quantified in detail from the observed changes in crystallographic orientation of the grains. Figure 2(a) shows a grain map reconstructed from the LabDCT scan of the copper particle sample at the initial step. Each grain is represented by a cube describing its position (centroid) and its crystallographic orientation, in relation to the absorption CT image of the sample. The imaged volume contains 114 particles comprising 212 detectable crystalline grains at the initial step, giving an average of 1.9 grains per particle. Changes in the grains have been tracked over all the time steps. Looking in more detail at the inverse pole figure representations of the grains within two regions of interest (RoI), one containing particles at the free surface (RoI1, Fig. 2(b)) and one fully contained within the sample (RoI2, Fig. 2(c)), it is clear that the grains rotate during sintering.

**Figure 3:**
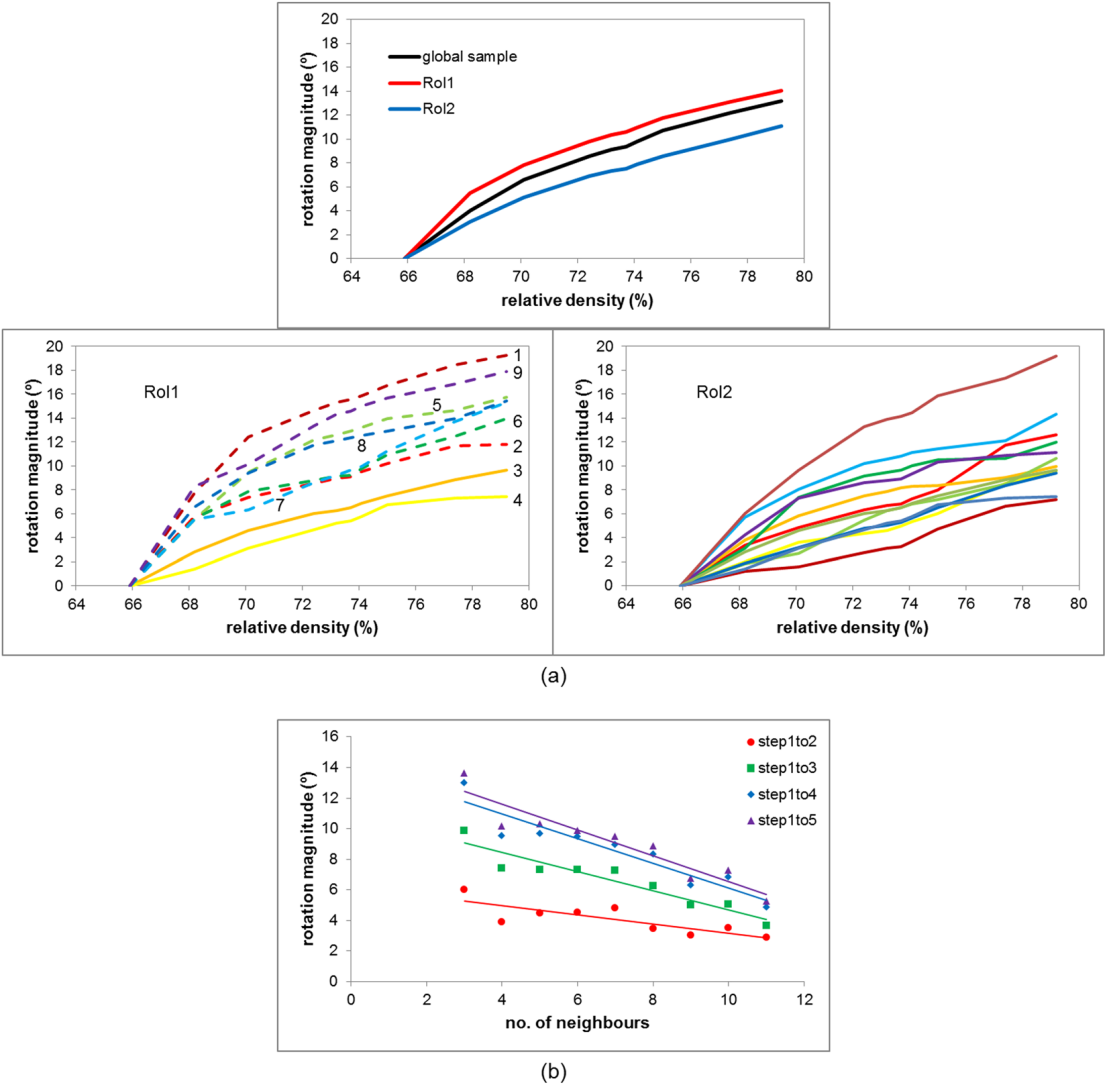
Quantification of particle rotations and effect of particle coordination. (**a**) Top: Plot of the average rotation of particles in the two RoIs illustrated in Fig. 2 and globally for the whole sample, as a function of relative density. The rotations are cumulative over the incremental sintering steps. Below: Cumulative rotations of individual particles in RoI1 (left, 9 particles) and RoI2 (right, 12 particles). The rotation profiles for particles in RoI1 are numbered corresponding to the particle numbers in Fig. 2(b). Particles in RoI1 that lie on the surface of the sample are shown with dashed lines. (**b**) Showing the mean particle rotations relative to the original arrangement as a function of the number of contacting neighbours, for the whole sample studied.

From the reconstructed LabDCT grain maps the magnitudes of rotation of the grains, and thus the particles, have been calculated between each sintering step. The rotation of a grain between two consecutive time steps is calculated from its misorientation between the two steps. While most particles contain more than one detectable grain (1.9 grains on average), for reasons of clarity only a single grain is displayed within each particle in Fig. 2(b) and (c). In three of the particles in RoI1 (particles 1, 3 and 4) two grains were measured and the agreement between their reorientations suggests that DCT can measure the reorientations to better than 0.05°. This also confirms that the particles themselves are rotating as solid bodies. Table 1 gives the measured cumulative total rotations over the first five sintering steps for all the measured grains in RoI1. In plotting the grain orientations on an inverse pole figure (Fig. 2(b) and (c)) it is evident that the scale of the particle movements between the two RoIs is different, with those particles in RoI1 (near the sample surface) showing larger changes in orientation. As an example, taking the particle in each RoI that shows a rotation close to the average rotation for all particles in that RoI, particle 8 of RoI1 marked in Fig. 2(b) rotates by 12.1° between sintering steps 1 and 5 while particle 11 of RoI2 marked in Fig. 2(c) rotates by 7.1°. Figure 3(a) shows the cumulative rotation of the particles as a whole and individually. From this it is clear that the rate of particle rotation slows as observed previously^25^ and suggests that particle rotations are greater for surface breaking particles than interior ones. RoI1 consists of both surface breaking and interior particles – while particles 5 and 9 (surface breaking, for example) marked in Fig. 2(b) rotate by 12.6° and 14.3° respectively between sintering steps 1 and 5, particles 3 and 4 (interior) rotate by 6.3° and 5.2°. Taken together, Figs 1 and 3(a) suggest a correlation between the observed difference in particle rotation between the two RoIs and their relative densifications (see inset of relative density plot in Fig. 1). While their relative densities eventually approach the same value, RoI1 densifies more rapidly over the initial steps, which correlates with larger particle rotations in this RoI. Figure 3(b) supports the theory that the degree of rotation is related to the coordination number of the particle, with those particles at the surface (e.g. particles 5 and 9 in RoI1) therefore rotating to a greater extent because of the smaller number of contacting particles (see also Table 1). The influence of particle coordination becomes more significant as sintering progresses, as evidenced by the rate of decrease of particle rotation with number of contacting neighbours. The average number of contacting neighbours shows a small increase over these first 5 time steps, from 6.5 to 6.7, reflecting the fact that new particle contacts are being created as sintering progresses. This might be a contributing factor in the observed deceleration in the rate of increase of particle rotation with sample densification (Fig. 3(a)).

**Table 1:** Cumulative rotations of individual particles in RoI1 over the first five sintering steps.

Particle No.	Rotation magnitude (°)		Number of Interparticle contacts
1	15.33	15.33	8
2	8.95		7
3	6.28	6.24	8
4	5.19	5.20	9
5	12.60		5
6	9.18		6
7	9.21		7
8	12.10		4
9	14.31		4

**Figure 4:**
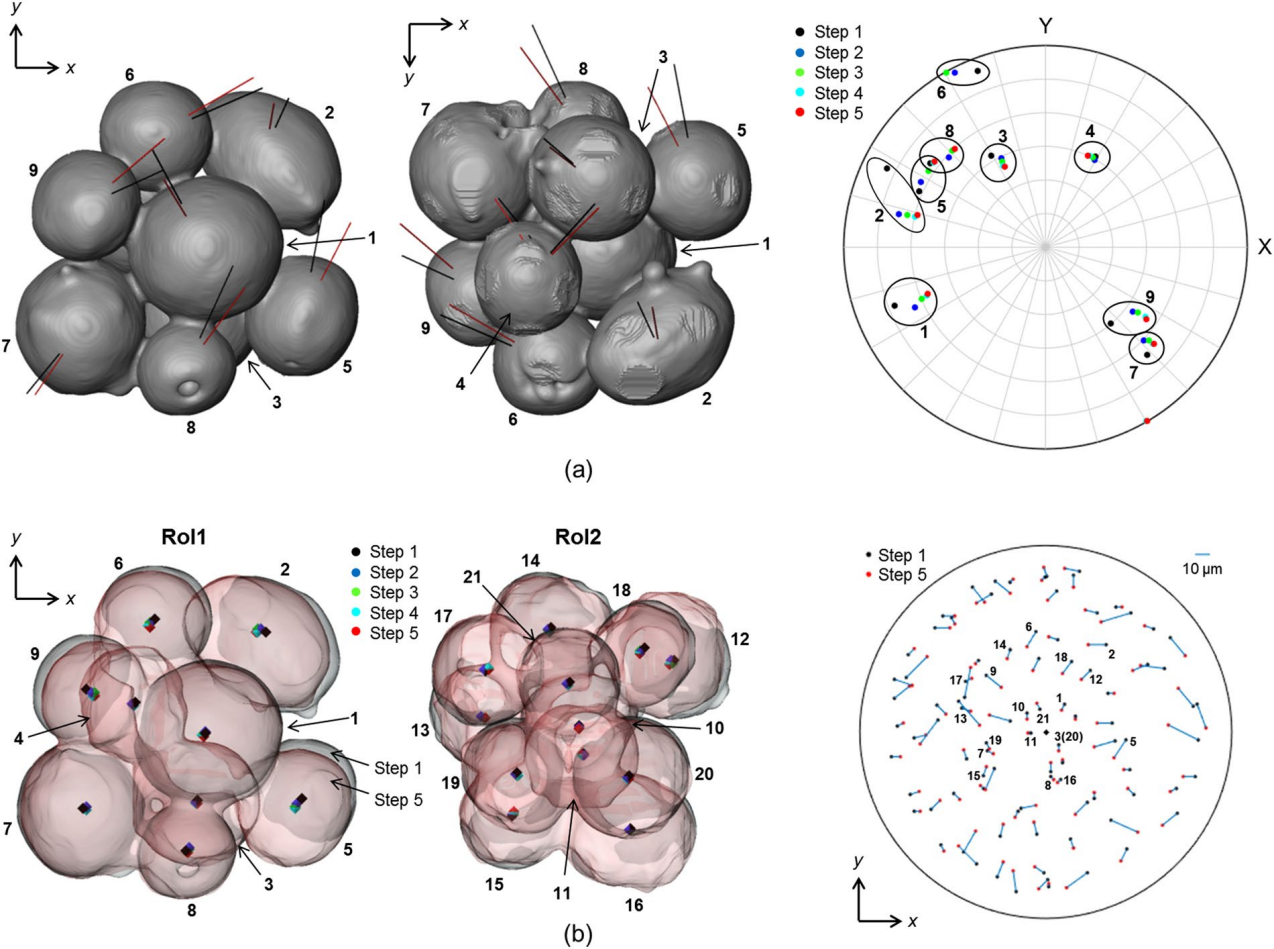
Visualization of particle rotations and their rotation ‘paths’ and particle translations. (**a**) Left and centre images: 3D isosurface rendering of the particles in RoI1 with vector lines representing the rotation axes of the grains within the particles (related to Fig. 3(b)) for step 1 (black lines) and step 5 (red lines). Note: the images represent different views (from above and below, respectively). Right-hand image: Spherical projection onto which the rotation axes are plotted for the 9 particles for each of the steps 1 to 5 (distinguished by the different colour labels, as indicated). Note: antipodal symmetry is applied, with the axes all plotted in the upper hemisphere. (**b**) Left and centre images: Plan views (transparent 3D surfaces) of RoI1 and RoI2 each showing sintering steps 1 and 5 overlayed to illustrate the changes in particle positions. Indicated within each particle are the measured particle centres at each step 1 through to 5. Right-hand image: Planar projection showing particle centre trajectories in the horizontal plane for all particles in the sample.

Figure 4(a) visualizes the rotation of each particle in RoI1 relative to its neighbours, demonstrating the cooperative rotations of the particles within the group. The rotation ‘paths’ of the particles are plotted for each of the steps 1 to 5, where the rotation axes of the grains are plotted in a spherical projection. In most cases the rotation paths are not linear trajectories, particularly between steps 1 and 2 (black and blue dots) suggesting that each particle ‘gyrates’ rather than rotates about a single axis as sintering progresses. While assemblies of particles are not observed to rotate as a single body, to a certain extent some particles rotate cooperatively (rather like two spheres rolling in contact with one another), however there are locations where the rotations are uncorrelated suggesting some sliding. Particle rearrangement is also characterized by particle translations as well as rotations. Particle translations are visualized in Fig. 4(b), measured and quantified using the conventional absorption CT data from the change in particle centres through sintering steps 1 to 5. Analogous to the particle rotation behavior, those particles in RoI1 show larger translations, an average of 8.3 µm in the horizontal plane compared to an average of 5.4 µm for particles in RoI2, suggesting that particle translations are also greater for surface breaking particles than interior ones.

### Grain growth

**Figure 5:**
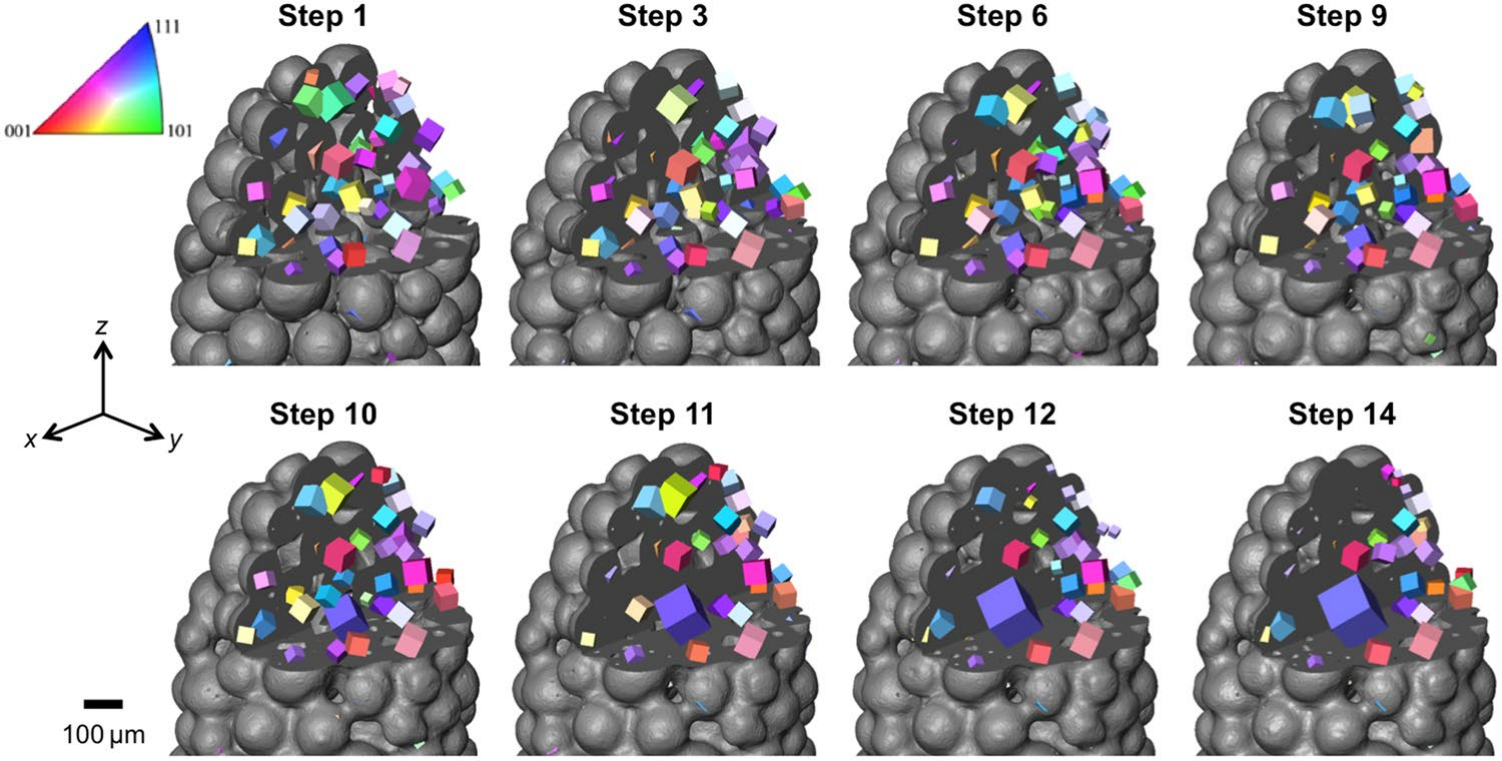
Microstructural evolution, both morphological and crystallographic, during densification of the powder body. Time-series showing the absorption tomography volumes (black and white surface representation) of the copper particle sample for several steps through the sintering sequence. The crystallographic microstructure within the sectioned region is revealed, allowing grains to be tracked through the time steps. The colourmap represents crystallographic orientation according to inverse pole figure; the cubes are scaled by 0.5 according to the respective grain size to allow for easier visualization of all the grains in the image.

**Figure 6:**
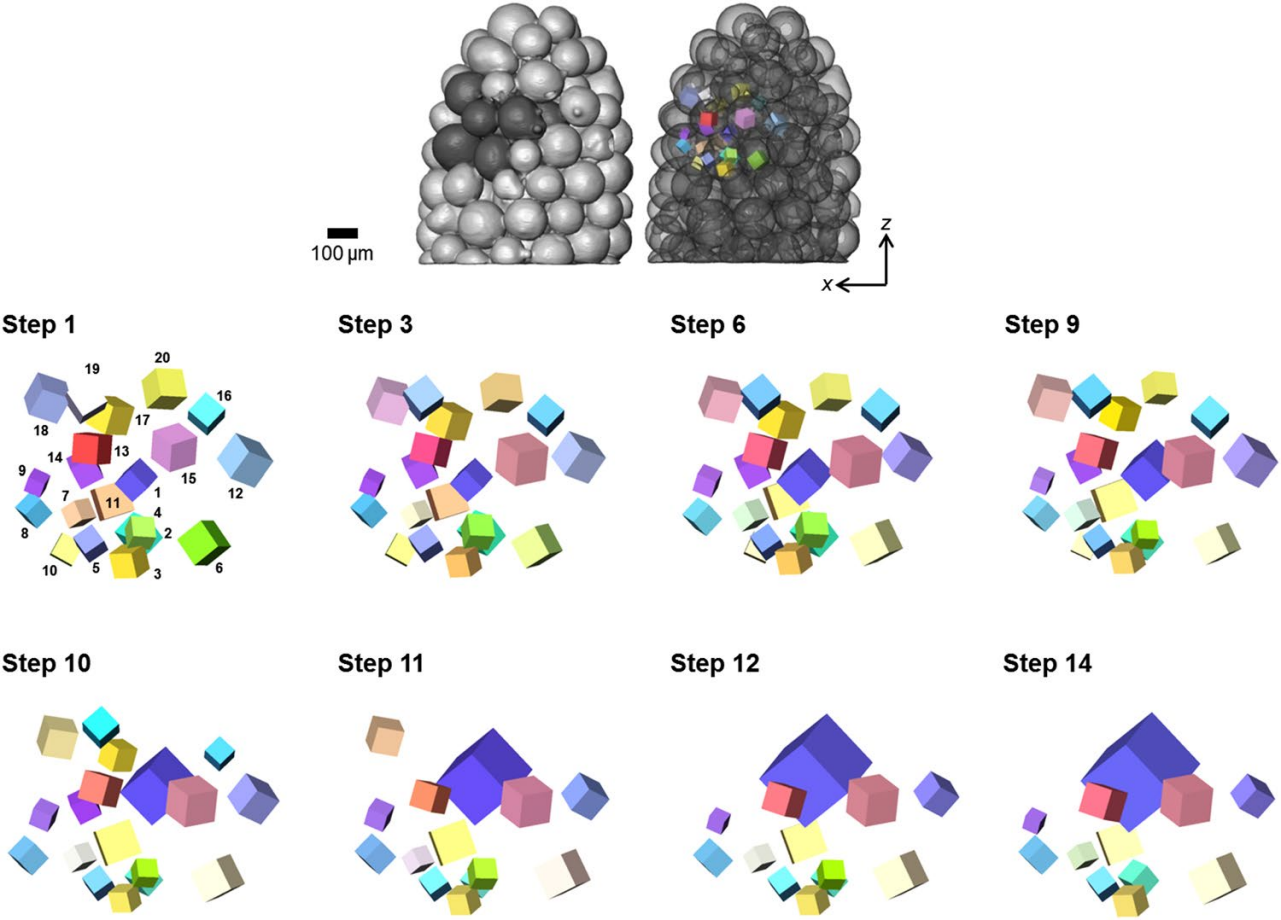
Tracking grains through a time-series of sintering steps. Illustrated above (in dark grey, left image) is a RoI (contains 12 neighbouring particles) in relation to the full sample. Illustrated below is the time evolution of the 20 grains within this RoI, (each numbered in step 1 (bottom)).

**Figure 7:**
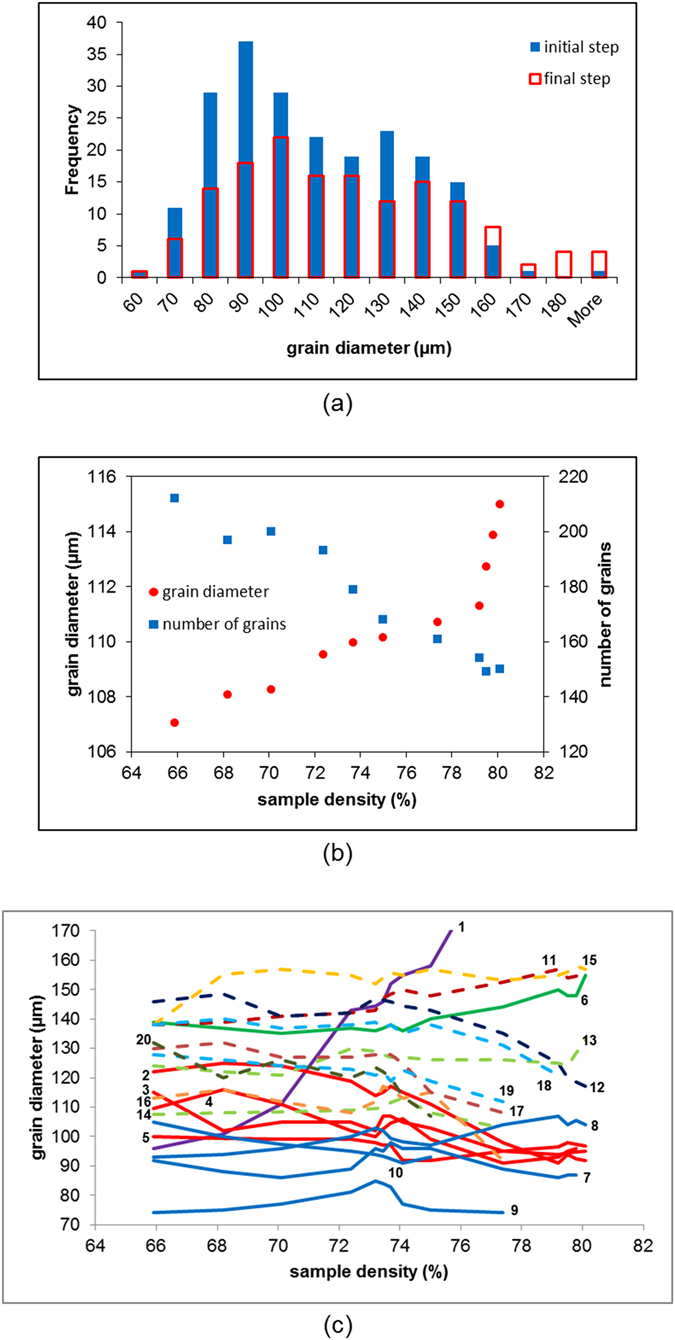
Quantification of grain growth and shrinkage. (**a**) Histograms showing grain diameter distributions at the initial and final sintering steps. (**b**) Showing both the average grain diameter and the number of grains through the sintering process, as a function of sample density. (**c**) Showing grain diameter profiles, as a function of sample density, for each of the grains in the local RoI of Fig. 6. The numbers correspond to the number labels indicated on the image of step1 in Fig. 6. Profiles with the same line colour correspond to grains within the same particle. Note: The profile for grain 1 is shown only up to 76% sample density – this grain continues to grow through to 80% sample density, reaching a size of approximately 310 µm.

Figure 5 shows the microstructural evolution of the sample, both morphological and crystallographic, for selected steps through the full sintering process. The number of grains detected decreases from 212 to 150, this reduction is clearly illustrated by the significant growth of certain grains such as that coloured purple in Fig. 5. This growing grain is accompanied by the dissolution of grains in its immediate vicinity, as is observed most significantly between steps 10 and 11 in Fig. 5. In order to visualize this competitive growth Fig. 6 tracks the grains in a small RoI centred on the fast-growing grain (labeled No. 1). This RoI comprises the nearest neighbour particles, and thus nearest neighbour grains, to the particle containing the growing grain. There are also examples of small grains that are eliminated over the sintering sequence. The histogram in Fig. 7(a) compares the grain diameter distributions in the full sample for the initial and final sintering steps. It is noteworthy that grains smaller than 60 µm are not detectable. The distribution for the final step shows a significant increase in the number of grains larger than 150 µm accompanied by a reduced number of grains having diameters below 150 µm. Clearly competitive grain growth is occurring, this is probably driven both by a reduction in grain boundary energy facilitated by the movement of particularly mobile grain boundaries. Work is ongoing, in terms of the ability to reconstruct the grain boundaries using the technique, so as to examine this. It is perhaps not surprising to note that as the rate of pressureless densification slows as 80% density is reached the rate of grain growth as a function of densification increases (see Fig. 7(b)). The growth and shrinkage of individual grains is quantified in Fig. 7(c) which plots the grain diameters as a function of sample density for each of the grains labeled 1 to 20 in step 1 of the small RoI in Fig. 6. Grain 1 grows to a size approximately double that of the next largest grain, while several other grains show limited growth and the remainder either shrink or are consumed entirely. Such measurements pave the way for direct comparison to theory/models (e.g. ref. 29) without having to rely on statistical measures of grain size distribution. In this context technique development work is ongoing that will enable the reconstruction of grain boundaries in the near future.

## Discussion

The development of a laboratory-based diffraction contrast tomography modality (LabDCT) has enabled in-depth studies of temporal changes in crystallographic grain structure during ‘4D’ or time-lapse studies of microstructure evolution. Here it has been used to follow the processes that occur during sintering of an ideal copper powder, non-destructively and in three-dimensions. The morphological changes have been followed through conventional absorption tomography, quantifying the development and growth of interparticle contacts between neighbouring particles and their translations. At the same time, diffraction contrast tomography has enabled particle rotations occurring during the sintering process to be inferred from measurements of the crystallographic orientations of the individual crystallite grains contained within the polycrystalline copper particles. While particle rearrangement during sintering has been measured and quantified previously using conventional absorption CT^2, 21^, it is this capability to link directly the crystallographic and grain microstructure information that creates a powerful tool for studying microstructure evolution. For example, one can link the relative densification measured from the absorption CT with observed changes in particle rotation (see Figs 1 and 3(a)), and also link to direct observations of grain growth and the measurement of growth dynamics of individual grains (see Fig. 7). The measurement of more than one grain within a single particle can give a useful measure of the accuracy of the LabDCT technique in measuring crystallographic orientation. The consistency between such grains is better than 0.05° between successive steps, suggesting a measurement accuracy of <0.05° in the determined grain reorientations and indicating that even small grain rotations can be measured. In comparison, the angular resolution of the electron backscatter diffraction (EBSD) technique for measuring grain orientation is in the order of a few tenths of degrees^30^. In addition, studying temporal changes within the bulk of one and the same sample, such as the dynamics/kinetics of grain growth for example, is precluded with the EBSD technique making direct interpretation very difficult since the evolution of the grain structure can’t be followed directly. This is due to the need for destructive serial sectioning in order to extend to the third dimension the 2D grain orientation maps recorded from the sample surface, typically using a focused ion beam (FIB, 3D-EBSD), though higher spatial resolution is achieved, albeit for relatively small volumes^31^.

According to^26^, based on a validation study performed on a β-titanium alloy, the LabDCT technique can accurately capture larger grains but doesn’t detect grains below 40 µm. The FIB section in Fig. 1 reveals that most grains are greater than 40 µm in size and indeed a few smaller than this value which would not be detected. It is noted that here grains smaller than 60 µm are not detected by LabDCT, neither in the initial sintering step nor in the final step (see Fig. 7(a)). The scattering power of the material, the flux density of the X-ray source and the sensitivity of the X-ray detector system all influence this value. While the scattered intensity or signal strength in the measured diffraction spots is proportional to the grain volume and to the third power of the grain radius, the detection limit in grain size is determined by the signal to background noise in the diffraction area of the detector. This is, consequently, influenced by the exposure time used during collection of the diffraction patterns. In fact, a second LabDCT scan of the sample at the final sintering step was subsequently recorded with an exposure time double that of the scan recorded in the time-series sequence described here. While the grain size distribution is very similar between these two scans, the scan with the longer exposure time detected 5 (smaller) grains in the 40–60 µm range. While it is acknowledged that smaller grains are detected in this way, in the current time-series sequence there remains at least one grain detected in each particle, thus allowing its reorientation (and the particle rotation) to be determined, and with consistency in the measurement where there are two grains in a particle. Furthermore, the ability to keep the total scan time as short as possible had the advantage of being able to perform a detailed time-series of 15 time steps within a practical timescale, taking into consideration maintaining stability of experimental conditions throughout the complete study, e.g. furnace heating, LabDCT measurements.

The degree to which a particle rotates is observed to be dependent on the number of contacting neighbours (see Fig. 3(b) and Table 1). As the number of particle contacts increases a particle finds its rotation increasingly restricted. Some rotations appear to be cooperative, rather like balls rolling over one another, while others are independent of nearest neighbours requiring particle sliding/shear at the contact grain boundary. This is assumed to be due to, as a driving force, the minimization of the total grain boundary energy of a particle in its 3D network. Grupp *et al*.^2^ found particles to roll with respect to their interparticle contacts but also to rotate by much larger angles around their own centres. They concluded that the crystallographic misorientation energy is strong enough to overcome the resistance due to the firm bonds between contacting particles allowing for grain boundary sliding. Identification of contacting grain neighbors through improvement to the analysis routines currently being implemented will allow further in-depth investigation into any potential role of misorientation at the sliding contact grain boundaries between particles as they rotate.

In tracking microstructure evolution during densification of the sample, the LabDCT technique has also provided insights into the grain growth processes occurring. Currently, we are only able to track the orientation and approximate size of each grain. Development of the analysis routines is in progress that will enable us to reconstruct grain shape information, providing more insights into the nature of the grain boundary movements responsible for grain growth at the later stages of sintering, both within particles and from particle to particle. Such information will also provide an indication as to those grain boundaries that are most mobile to better understand the competitive aspects of grain growth. Grain boundary mobility is sensitive to several factors including the misorientation between two neighbouring grains^4^. Grain boundary energy also depends on the misorientation at the grain–grain contact and certain orientations have been observed, based on atomistic simulations, to give preferred behavior^32^. From a random formation of grain pairs, depending on the misorientation, torque arises from the gradient in surface energy with orientation, slowly rotating the grain pair toward a point of lowest energy. Cases of initially low misorientation angles resulted in coalescence (the grains rotated to zero misorientation). Grains of large size difference are also observed to favour coalescence^33^. The ability to map the grain boundaries in 3D during evolution of the microstructure will enable us to analyse in more detail their development and mobility during sintering, in light of misorientation between grain neighbors for example. An approach to extract grain boundary/grain shape information from LabDCT datasets has been identified and we look forward to applying this in the near future.

## Methods

### Material

Spherical polycrystalline copper (>99.3% purity) powder particles, having a mean particle diameter of 120μm and manufactured by air-atomisation, were supplied by Makin Metal Powders (UK) Ltd. The initially loose Cu particles were contained within a quartz-glass capillary of 0.7mm internal diameter. Sintering was performed at a temperature of 1050 °C using a tube furnace under inert gas atmosphere. The sample was pre-sintered at 800 °C for 10 minutes to stabilize the grain structure of the particles and to encourage recrystallization and grain growth such that the grain size was increased from the 5 µm in the as-received powder in order to give grains larger than the minimum grain size currently detectable by LabDCT (~40μm^26^). Metallographic analysis of the powder particles after the pre-sintering step revealed the grain size to be ~40 µm with ~5 grains per particle. While the pre-sintering step did cause a small degree of sintering of the particles, all subsequent observations of microstructure evolution, such as particle rotations, are measured relative to this initial step.

### LabDCT measurements

LabDCT was carried out on a ZEISS Xradia 520 Versa X-ray microscope equipped with the GrainMapper3D™ analysis package. The source–sample and sample–detector distances were both set to 12mm, thereby exploiting the 1:1 distance ratio of the source and detector to benefit from the Laue focusing effect^26, 28^. The effective pixel size was 1.7 µm. An accelerating voltage of 120 kV and current of 75 µA were used to acquire 180 diffraction pattern images around a 360° rotation of the sample in steps of 2° with an exposure time per image of 60 s, resulting in a DCT scan time of 3 hours. 720 projection images each of 2 s exposure were acquired for the absorption tomography.

The sintering process was interrupted 15 times in order to capture two CT scans each time (an absorption scan and a DCT scan). Other experiments have shown that this interruption does not significantly affect the sintering kinetics. For each sintering step the sample was heated from room temperature with a heating rate of 100 °C min^−1^. After each sintering step, in order to perform the LabDCT measurements, the sample was removed from the furnace whilst at temperature, i.e. there was no cooling of the sample/furnace to room temperature prior to sample removal. For absorption CT, an aperture placed between the source and sample restricts the size of the direct X-ray beam, illuminating the sample only in the central region of the detector and leaving the outer part dark (see^26, 28^). For the DCT, a beam stop was used to attenuate the direct X-ray beam transmitted through the sample in order to be able to collect high fidelity diffraction patterns^26, 28^. Such images were used to reconstruct the orientations of grains and their positions in relation to the sample reconstruction mask. Grain map reconstruction^26^ was performed using GrainMapper3D™ analysis software from Xnovo Technology.

The absorption images from the sequence of sintering steps were aligned with one another using Avizo visualization and analysis software, based on 3D surface reconstructions of the particle microstructure. The same alignment transformations were applied to the corresponding DCT grain map reconstructions ensuring their alignment between sintering steps. Tracking of grains through the sintering time series, and the calculation of changes in grain orientation and grain centroid positions, was performed using GrainMapper3D™ Registration Tool.
